# Comparison of DNA repair and radiosensitivity of different blood cell populations

**DOI:** 10.1038/s41598-021-81058-1

**Published:** 2021-01-28

**Authors:** Daniel Heylmann, Viviane Ponath, Thomas Kindler, Bernd Kaina

**Affiliations:** 1 Institute of Toxicology, University Medical Center, Johannes Gutenberg-University Mainz, Obere Zahlbacher Straße 67, 55131 Mainz, Germany; 2 Department of Medical Oncology and Pneumology, University Medical Center, Johannes Gutenberg-University, Mainz, Germany; 3 Present Address: Rudolf Buchheim Institute of Pharmacology, Justus Liebig University, Giessen, Germany; 4 Present Address: Center for Tumor Biology and Immunology, Institute for Tumor Immunology, Philipps University, Marburg, Germany

**Keywords:** Medical research, Immunology, Cell death and immune response, Immune cell death

## Abstract

Despite the frequent use of ionising radiation (IR) in therapy and diagnostics and the unavoidable exposure to external radiation sources, our knowledge regarding the radiosensitivity of human blood cell populations is limited and published data, obtained under different experimental conditions, are heterogeneous. To compare the radiosensitivity of different hematopoietic cell populations, we set out to determine the responses of cells obtained from peripheral blood of healthy volunteers under identical conditions (resting, non-stimulated cells). First, we measured the radiation response of T cells (Treg, Th, CTL), B cells, NK cells, CD34+ progenitor cells and monocytes obtained from peripheral blood and monocyte-derived macrophages (Mph) and immature dendritic cells (iDC) ex vivo and show that T and B cells are highly sensitive, starting to undergo apoptosis following IR with a dose as low as 0.125 Gy. Importantly, there was no clear threshold dose and cell death/apoptosis increased up to a saturation level with a dose of 2 Gy. The sensitivity decreased in the order of T cells > NK and B cells > monocytes > macrophages and iDC. The data confirm a previous report that Mph and iDC are radiation-resistant compared to their progenitor monocytes. Although non-stimulated T and B cells were highly radiation-sensitive compared to monocytes and macrophages, they were competent in the repair of DNA double-strand breaks, as shown by a decline in γH2AX foci in the post-exposure period. CD34+ cells obtained from peripheral blood also showed γH2AX decline post-exposure, indicating they are repair competent. Granulocytes (CD15+) did not display any γH2AX staining following IR. Although peripheral blood lymphocytes, the main fraction are T cells, were significantly more radiation-sensitive than monocytes, they displayed the expression of the repair proteins XRCC1, ligase III and PARP-1, which were nearly non-expressed in monocytes. To assess whether monocytes are depleted in vivo following IR, we measured the amount of T cells and monocytes in cancer patients who received total-body radiation (TBR, 6 × 2 Gy). We observed that the number of T cells in the peripheral blood significantly declined already after the first day of TBR and remained at a low level, which was accompanied by an increase in the number of γH2AX foci in the surviving CD3+ T cell fraction. In contrast, the number of monocytes did not decline extensively, reflecting their radiation resistance compared to T cells. Monocytes also showed an accumulation of γH2AX foci in vivo, but the levels were significantly lower than in T cells. CD56+ NK cells displayed a response similar to T cells. The data support the notion that unstimulated T cell subfractions are nearly equally radiation sensitive. There are, however, remarkable differences in the radiation sensitivity between the lymphoid and the myeloid lineage, with lymphoid cells being significantly more sensitive than cells of the myeloid lineage. In the myeloid lineage, macrophages and iDCs were the most radio-resistant cell types.

## Introduction

Suppression of hematopoiesis is a severe toxic side effect of radiotherapy (RT) in cancer patients^1^. It also impacts healthy individuals since humans are constantly exposed to external radiation caused by natural background sources, diagnostic procedure or, at the extreme end of the spectrum, nuclear accidents^2^. In cancer radiotherapy, which is applied in ~ 60% of cancer treatments, high doses of 1.8 to 2.0 Gy are usually used per fraction. Although high precision irradiation results in improved selective tumour cell killing, immunosuppression can be caused by affecting immune cells that circulate in blood vessels in the irradiated tumour field. Immunosuppression may also result from death of cytotoxic T cells and other immunocompetent cells that have infiltrated the tumour tissue. It is conceivable that this eventually leads to an attenuated tumour-directed local immune response. Of note, high dose radiation is also used for eradicating immune cells, including residual tumour cells, prior to bone marrow or hematopoietic stem cell transplantation, where several fractions of doses of 2 Gy are applied^3^. Low dose irradiation with single doses of 0.1 up to 0.5 Gy is frequently used for treatment of benign diseases, notably degenerative and inflammatory syndromes^4,5^. In this case, the curative effect is thought to rest mainly on the modulation of the immune response in the chronic inflammatory tissue^5^. Although high and low dose radiation therapy is frequently applied worldwide, our knowledge regarding the sensitivity of human blood cell populations is limited. Also, radiation exposure for diagnostic purposes and following external exposures demands a precise insight into the radiation sensitivity of cells of the hematopoietic system. The current knowledge on radiation sensitivity of hematopoietic cell populations has been reviewed extensively^6^ and gaps in our knowledge are obvious.

The immune response rests on a balanced activity of B cells, the different T cell populations such as cytotoxic T cells (CTL), regulatory T cells (Treg) and T helper cells (Th), neutrophils and other granulocyte populations, and monocytes (Mo) that generate macrophages (Mph) and dendritic cells (DCs). Previously, we have shown that monocytes irradiated ex vivo are more radiation-sensitive than macrophages and DCs derived from them by cytokine treatment, but a comparison with other blood cell populations was lacking in this study^7^.

Here, we measured the death responses of different cell populations obtained from peripheral blood of healthy volunteers in vitro, using annexin V flow cytometry and γH2AX assay. We assessed the apoptotic response of CTL, Treg, Th, B and NK cells and compared them with granulocytes, monocytes, macrophages and iDCs. The data revealed clear differences in cell population responses, which are discussed in the context of previously published data. We also measured in a pilot study the radiation toxicity and γH2AX levels in patients upon total-body radiation (TBR) and compared the results with the data obtained in vitro.

## Results

**Figure 1:**
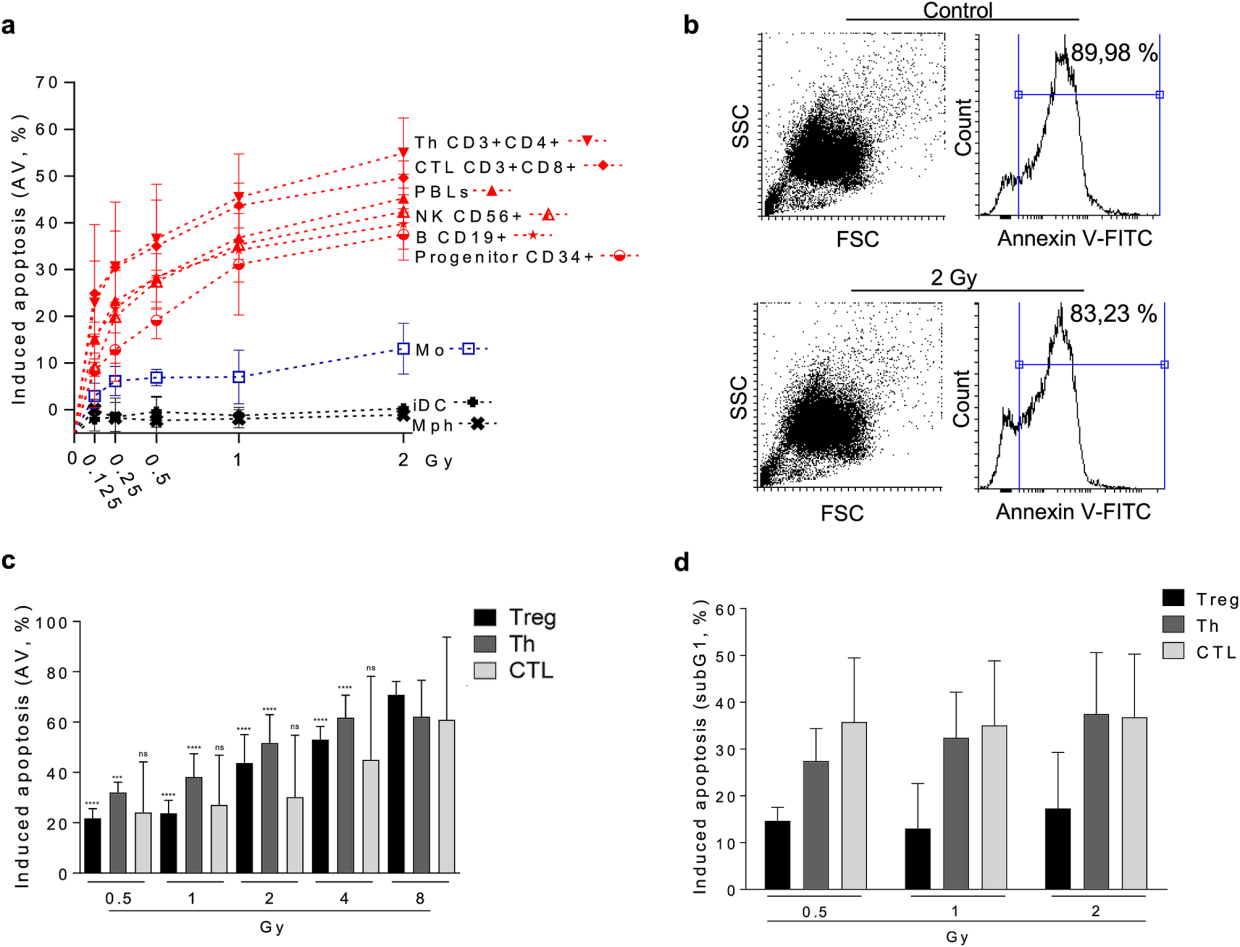
Radiation-induced cell death of different blood cells from the myeloid and lymphoid lineage. (**a**) After radiation treatment, significantly more cells of the lymphoid lineage (Th, CTL, NK cells, B cells and CD34 progenitor cells) undergo radiation-induced cell death compared to monocytes and the radioresistant iDC and Mph. Apoptosis was determined by annexin V-flow cytometry 24 h after treatment. Mean value, SD, n = 3. Data for Th, CTL, PBLs and CD34+ are from ref^8^ where they were shown in a different context. (**b**) Granulocytes showed already a high basal level of cell death, independent from radiation treatment, as revealed by annexin V-staining. (**c**) Radiation-induced cell death of Treg, Th and CTL purified with magnetic beads. Data for 0.5 and 1 Gy are from ref^8^. At 0.5 Gy, regulatory T cells are more resistant to IR compared to Th. CTL showed high interexperimental variations. Mean value, SD, n = 3. The induced frequencies were significantly above the control levels (which are shown in Fig. S1c), ns, not significant; *p < 0.05, **p < 0.01. (**d**) Radiation-induced apoptotic DNA fragmentation in Treg, Th and CTL as analysed by the subG1-assay. There was a tendency for Treg to undergo less DNA fragmentation than Th and CTL. Mean value +/− SD, n = 3–4.

A comparison of different blood cell populations as to their dose–response following irradiation is shown in Fig. 1a. The data revealed that cells of the lymphoid lineage are more sensitive to IR than myeloid cells. Thus, already after 0.125 Gy a significant fraction of T and B cells was annexin V (AV) positive, indicating that death occurred through apoptosis in the dose range between 0.125 and 2 Gy. Total peripheral lymphocytes (PBLs) were responding similar to T and NK cells (Fig. 1a). It should be noted that the spontaneous levels of AV positive T and B cells were in the range of 20 to 40%, which was taken into account in the calculation of induced frequencies (the total apoptosis levels are shown in Supplement Fig. S1a, for statistics see Fig. S1b). As previously reported, apoptosis is the major route of cell death in lymphocytes following IR. In contrast, following treatment with the crosslinking agent mafosfamide, a much higher amount of lymphocytes (about 80%) were found to be necrotic^8^, indicating activation of the apoptotic or necrotic pathway in lymphocytes occurs in an agent-specific way. CD34+ progenitor cells isolated from peripheral blood by Milteny beads displayed a slightly lower radiation sensitivity than B, T (Th, CTL) and natural killer (NK) cells. We should note that these CD34+ cells were cultivated in cytokine supplemented medium in order to ensure high survival of the population. Freshly isolated CD34+ cells not cultivated in cytokine supplemented medium displayed a higher level of basal and induced apoptosis and, therefore, were difficult to compare with the other cell populations in the same setting. In the myeloid lineage, monocytes proved to be clearly more sensitive than macrophages and immature DCs, which were highly resistant (Fig. 1a). This is in accordance with previous reports^6,7^.

Granulocytes were obtained from peripheral blood and investigated separately. They show, after one day of ex vivo cultivation, an extremely high level of apoptosis, which was not further enhanced in the IR-treated fraction (Fig. 1b for representative plots). As reported previously^9^, granulocytes do not undergo IR-induced cell death, but die spontaneously during the incubation period, which is  likely triggered by an intrinsic cell death programme.

In another experimental setting, we compared the minor fraction Treg (CD4+, CD25+) with the main T cell subfractions Th (CD4+) and CTL (CD3+ CD8+). In this case, cells were purified from peripheral blood of healthy donors by magnetic beads, incubated in medium, irradiated and continued to be cultivated for 72 h. As in the other settings, the cells were not stimulated to divide. Apoptotic cell death was measured by AV-flow cytometry. Radiation-induced apoptosis of Treg, Th and CTL increased as a function of dose up to 8 Gy. The induced cell death level was only slightly different between Th, Treg and CTL, and statistically significant differences between the T cell populations were not observed (Fig. 1c and Supplement Fig. S1c). The only exception were Treg cells after 0.5 Gy, which showed significantly less induced cell death compared to Th (Fig. S1d). Overall, the data are in line with the dose–response curves shown in Fig. 1a, in which Th and CTL showed a nearly similar response. We also measured cell death by subG1 quantification. In this case, Treg were less sensitive than Th and CTL, although the differences between Treg and CTL were statistically not significant (Fig. 1d). Taken together, the data led us to conclude that Th, Treg and CTL represent the most radiation-sensitive cell types in the blood cell population. The high interindividual variation observed in blood samples from different donors did not allow a final conclusion as to whether Treg, Th and CTL differ significantly in their radiation sensitivity. Furthermore, we did not observe large and significant differences in cell death induction following radiation of NK and B cells compared to T cells. However, similar to T cells, NK and B cells are much more radiosensitive than monocytes, macrophages and iDCs (Figs. 1a and S1b).

**Figure 2:**
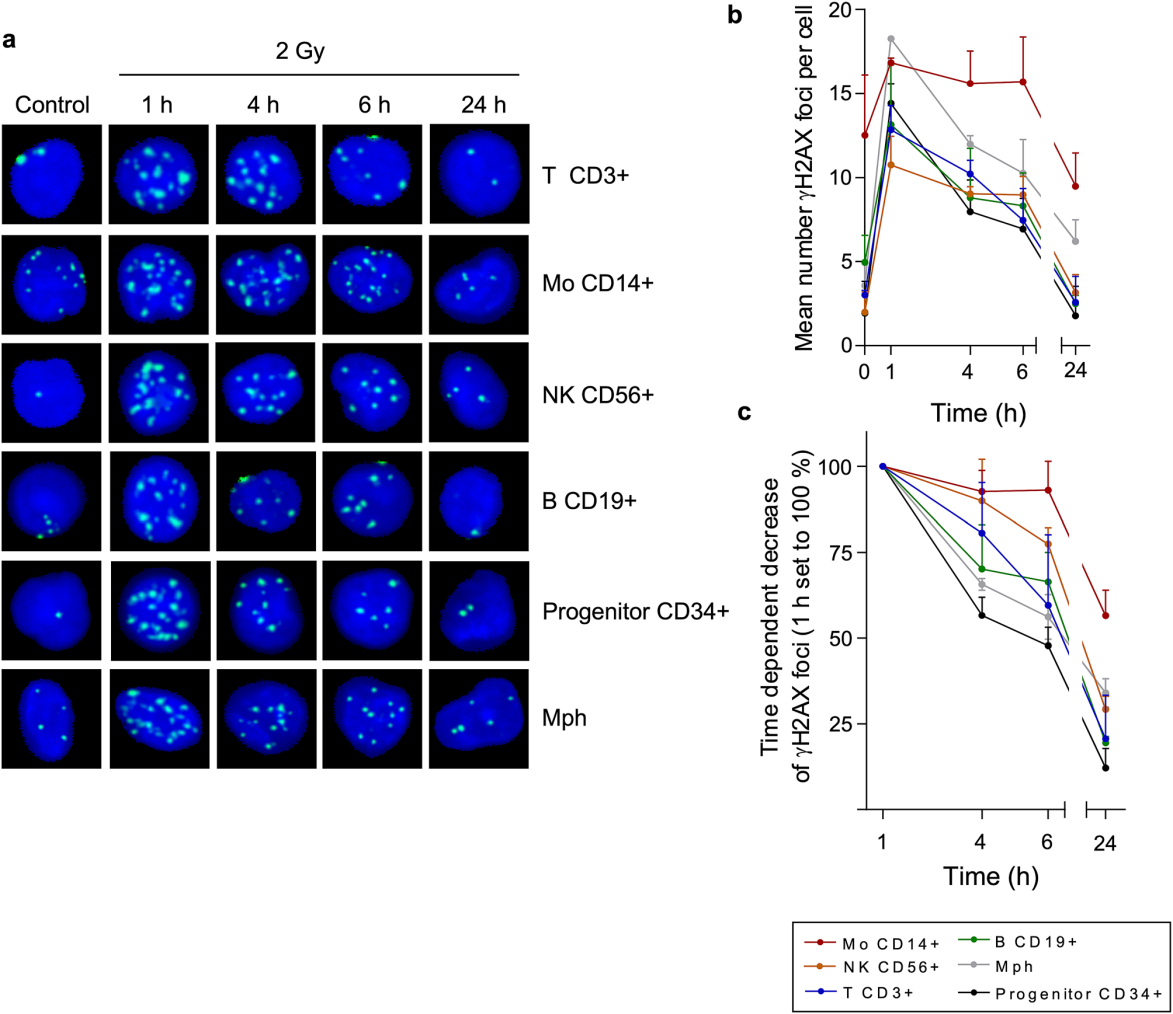
DNA repair kinetics of blood cells from the myeloid and lymphoid lineage assessed by γH2AX foci staining. (**a**) γH2AX foci determined by the metafer scanning system in different blood cell populations 1 up to 24 h after 2 Gy IR. After 1 h, there was a maximum of the γH2AX signal detectable, which declined time-dependently in all blood cells to the basal level. Nucleus, blue; γH2AX foci, green (**b**) Absolute number of γH2AX foci. Monocytes showed already a high level of γH2AX foci in the untreated control. Mean value, SD, n = 3 to 4. Cell counts per experiment (min–max): B cells, 300–314; NK cells, 299–307; T cells, 300–316; CD34, 56–305; CD14, 30–305; Mph, 238–303. (**c**) Relative amount of γH2AX foci (the 1 h value was set to 100%). NK cells, T cells, B cells macrophages and CD34 progenitor cells showed efficient DNA repair of IR-induced DNA damage obtained by γH2AX staining. Monocytes showed a high basal level (presumably due to lack of immediate-early repair) and up to 6 h a lack of foci decline.

The radiation sensitivities observed in the apoptosis assays prompted us to compare γH2AX foci formation (which is an accepted indicator of DNA double-strand breaks, DSB^10,11^) and their decrease with time (which is an indicator for DSB repair) in these populations. In Fig. 2a, representative examples of γH2AX foci are shown for different time points after irradiation. The quantitative assessment revealed that in all cell types γH2AX foci clearly declined in the post-exposure period of 6 h, except for monocytes (Fig. 2b, and in Fig. 2c normalised to the 1 h value). Thus, in T, NK, B cells, macrophages and CD34+ progenitor cells the remaining DSB level 6 h after treatment was about 50% of the induced level, which was not the case in monocytes. In the subsequent post-incubation period (up to 24 h) the foci level further decreased, which was also observed in monocytes. It thus seems that following exposure to a dose of 2 Gy monocytes are able to repair DSBs to some extent whereas at a higher dose level (5 Gy) DSB repair is strongly attenuated and appears to be saturated^7^. It should be noted that the basal level of γH2AX foci in monocytes was higher than in the other cell populations, which might be due to accumulation of DNA damage resulting from impaired DNA repair in this cell type.

**Figure 3:**
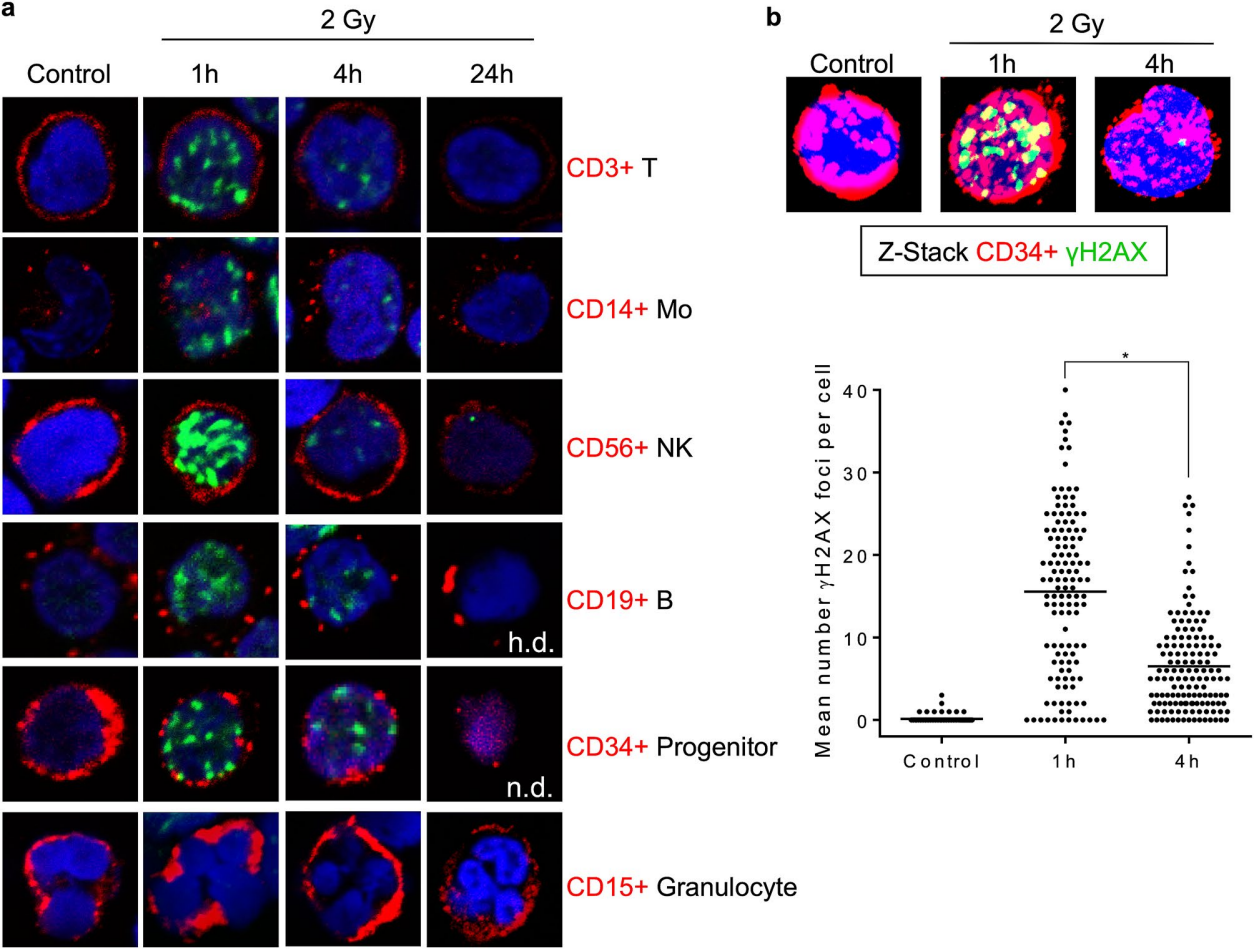
Staining of γH2AX (green) and CD surface markers (red) of different blood cell types purified from buffy coat. (**a**) Representative pictures of unirradiated cells (control) and samples 1 and 24 h after 2 Gy IR. A maximum of γH2AX foci accumulate 1 h after exposure to 2 Gy. γH2AX foci disappear 24 h after radiation implicating effective repair of IR-induced DNA double-strand breaks. CD19+ B cells are hardly detectable (h.d.) on the slide 24 h after treatment. Furthermore, no CD34+ progenitor cells could be found on the slide 24 h post-irradiation (n.d. not detectable). Granulocytes, shown in combination with the CD15 marker, did not show any IR-induced formation of γH2AX foci. (**b**) Quantification of γH2AX foci in CD34+ progenitor cells 1 and 4 h after 2 Gy IR. Representative 3 dimensional pictures (Z-stacks) recorded by the LSM are displayed above the graph. The decrease of γH2AX foci over time indicates an efficient repair of DNA double-strand breaks in CD34+ progenitor cells. n = 3, in total 84–142 cells per box. t-test, *p < 0.05.

In another experimental setup, cells were stained with γH2AX together with their corresponding surface markers and analysed by LSM. As shown in Fig. 3a, 1 h after radiation treatment γH2AX foci appeared, which were completely vanished 24 h later, indicating DSB repair was carried out. We should note that CD19+ B-cells and CD34+ progenitor cells were difficult to analyse 24 h after radiation treatment because of their strong depletion. For CD34+ cells, the γH2AX foci kinetic was analysed 1 and 4 h after 2 Gy in double-stained cells by LSM. The data (Fig. 3b, which shows representative images as Z-stacks and the quantification) are in line with the results obtained in the experiments shown above (Fig. 2c) and confirmed that CD34+ cells obtained from peripheral blood are able to repair IR-induced DSB. We should note that the basal level of γH2AX in the experimental series shown in Figs. 2 and 3 is different, with lower basal levels when evaluation occurred by LSM instead of the Metafer system, which is presumably due to different settings having impact on reducing the background staining. Interestingly, granulocytes (CD15+) did not show any γH2AX foci formation upon irradiation (Fig. 3a), which confirms a previous report^9^.

Monocytes were shown to exhibit attenuated DNA repair due to downregulation of key repair proteins^7,12–14^. This is confirmed in Fig. S2, showing the extremely low expression levels of ligase III, XRCC1 and PARP-1 in monocytes compared to PBLs. It is striking that monocytes, although they exhibit a repair defect, are significantly more radiation-resistant than peripheral blood lymphocytes including the different T cell populations (Fig. 1). The possible reasons will be discussed below.

**Figure 4:**
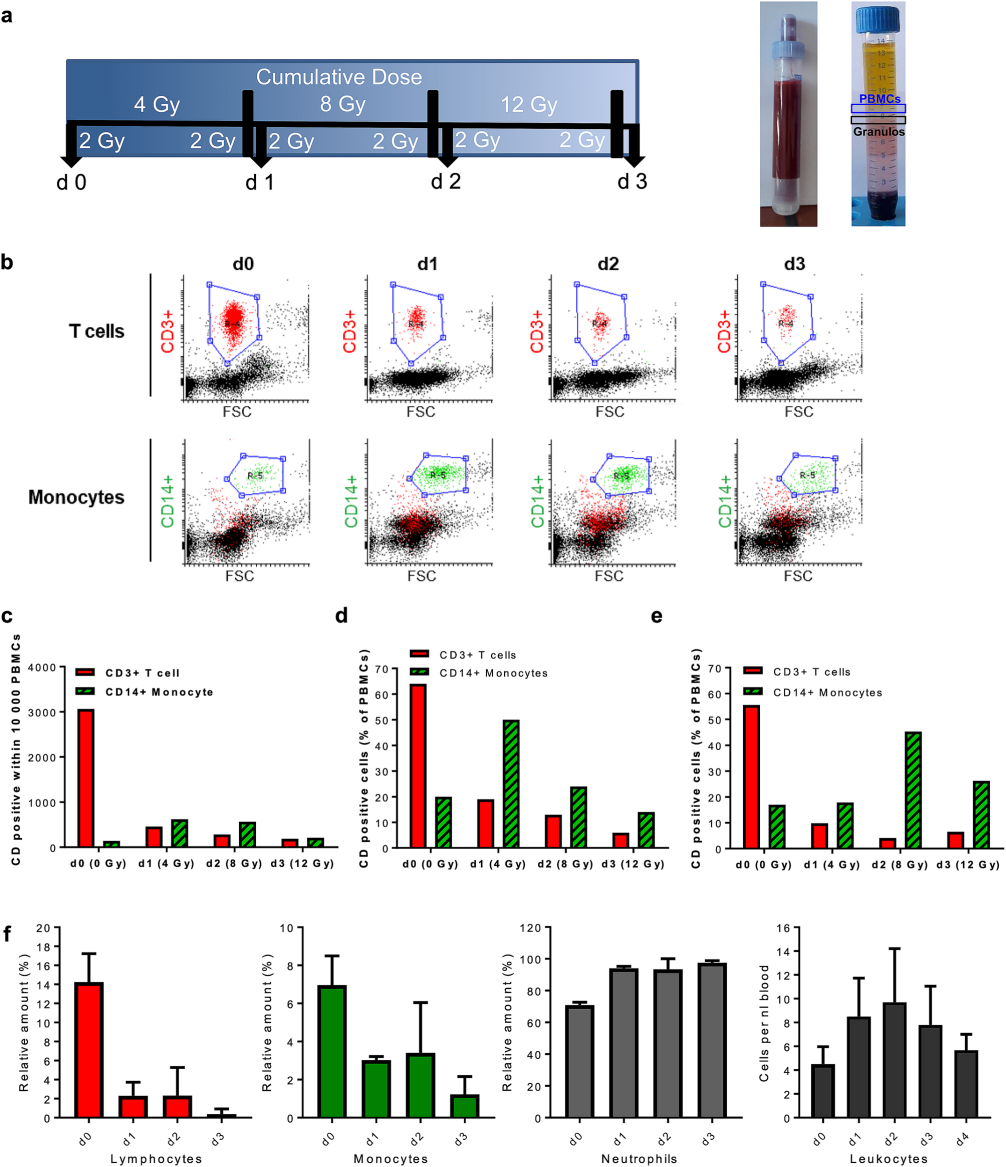
Treatment scheme of leukaemia patients, blood sampling during whole-body irradiation and influence of radiation on the number of T cells (CD3+) and monocytes (CD14+) in peripheral blood following total-body radiation. (**a**) Patients received a cumulative dose of 12 Gy, fractionated in 2 × 2 Gy per day. Blood sampling at day 0 (d0) occurred before the first 2 × 2 Gy fraction was administered (non-irradiated control). Blood samples on d1 and d2 were taken every morning before the patients were treated with further 2 × 2 Gy. The d3 blood sample marks the last day after cumulative 12 Gy of irradiation. Purification of PBMC and granulocytes was performed by polymorphprep™ density centrifugation. (**b**) Blood counts of CD3+ (T cells) and CD14+ (monocytes) cells in the PBMC fraction from one patient determined by flow cytometry. From day zero on, there was a clear decrease of T cells in the peripheral blood. Monocytes show at d1 and d2 a relative increase in the PBMC population. (**c**) Quantification of CD3+ T cells and CD14+ monocytes in PBMC of the patient indicated in (**b**). (**d**,**e**) Amount of CD3+ T cells and CD14+ monocytes determined in the PBMC samples obtained from two different patients. The determination occurred on stained slides by LSM (100 cells each). The data indicate a time-dependent decrease in the T cell number, while monocytes even displayed an accumulation at d1 and d2. (**f**) Relative amount of lymphocytes, monocytes and neutrophiles and total amount of leukocytes in the peripheral blood of patients during the TBR period. Data obtained from up to 8 patients are pooled and presented as mean +/− SD.

Is the radiation sensitivity of human blood cell populations determined ex vivo reflected in vivo? We addressed this question by measuring the amount of hematopoietic cell populations and their γH2AX levels in the peripheral blood of patients repeatedly treated with 2 × 2 Gy per day (cumulative dose 12 Gy) total-body radiation (TBR) (for the treatment setting see Fig. 4a). In Fig. 4b, flow cytometry counts obtained from one patient are presented. Their quantification revealed a significant decline in the amount of CD3+ T cells in the PBMC population while the number of monocytes were not reduced after cumulative doses of up to 12 Gy (Fig. 4c). This is compatible with what we observed in vitro, where monocytes showed significantly less cell death following IR than T cells (Fig. 1a). In Figs. 4d,e, the relative amount of T cells and monocytes in the PBMC population obtained from two different patients are shown. Again, there is a massive decrease of T cells during TBR, which was observed already on the first day after radiation treatment, whereas monocytes did not decrease in number. They even showed, after 1 and 2 days, a transient accumulation (Fig. 4d,e, respectively). We shoud note again that these experiments were performed with PBMC samples in which the granulocyte fraction had been almost completely removed by density centrifugation. If we assess the total blood counts in the patients (Fig. 4f), the number of lymphocytes drastically decreased, which occurred already 1 day after 2 × 2 Gy (by about 95%). The number of monocytes also declined, but clearly to a lesser extent (by about 50% on day one of TBR) while the amount of granulocytes (neutrophiles) and, concomitantly, the amount of total leukocytes increased. The relative increase of neutrophiles, compensating the decline of lymphocytes and monocytes, is compatible with their strong radioresistance^9^. It is also conceivable that during the TBR period their pool is filled up by a radioresistant progenitor, causing even an excess compensation. In summary, during TBR, we observed a strong decline in the number of lymphocytes and, to a clearly lesser extent, monocytes in the peripheral blood. The neutrophile population was not affected. This reflects the cell's radiosensitivity determined ex vivo.

**Figure 5:**
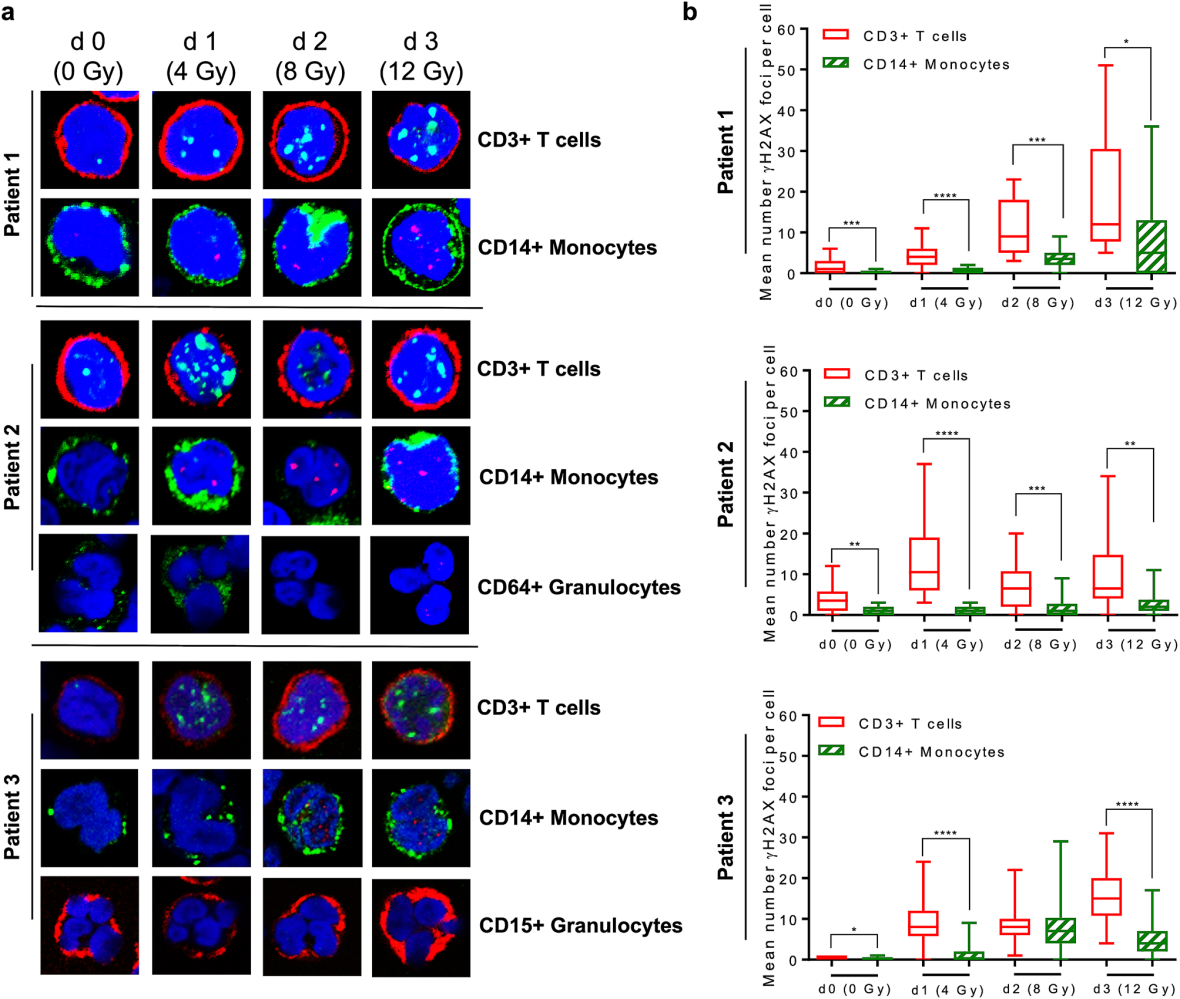
γH2AX staining in T cells (CD3+), monocytes (CD14+) and granulocytes (CD64+ or CD15+) isolated from patients during total-body radiation. (**a**) Representative images are shown of γH2AX foci (red or green) and CD surface marker (green or red) of PBMC at day 0 up to day 3 obtained from three different patients. Similar to what we observed for ex vivo irradiated granulocytes obtained from peripheral blood, no γH2AX foci could be detected in granulocytes upon exposure to ionising radiation in vivo. (**b**) Quantification of γH2AX foci in T cells and monocytes. In T cells significantly more γH2AX foci were induced following increasing cumulative doses of IR compared to monocytes. Depending on the yield and quality of the sample, 11 to 50 cells were counted per day and patient (Patient 1, 11 up to 20 cells; Patient 2, each data set 20 cells; Patient 3 each data set 50 cells). Box plots, t-test, *p < 0.05, **p < 0.01, ***p < 0.001, ****p < 0.0001.

**Figure 6:**
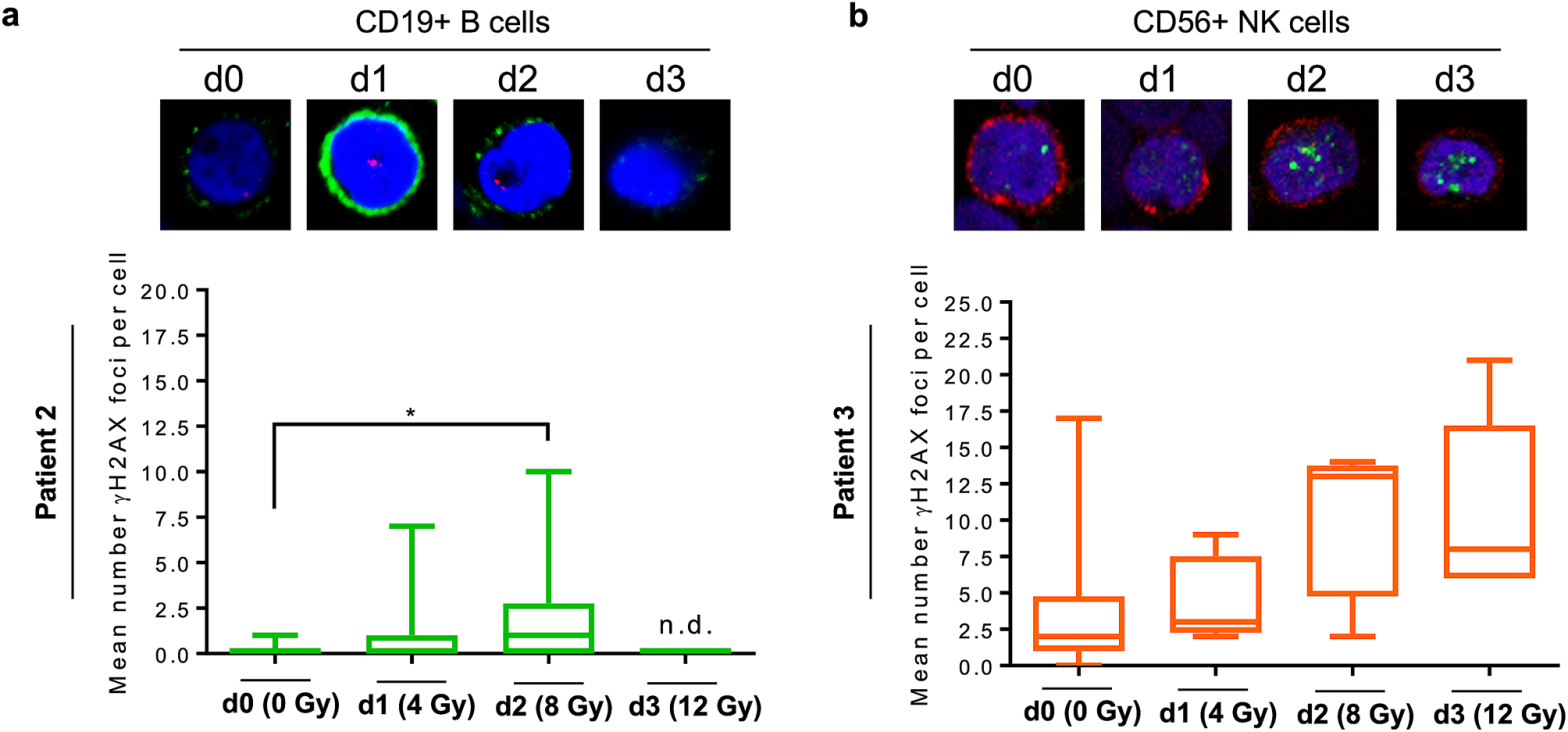
Quantification of γH2AX foci in (**a**) CD19+ B cells (CD19, green; γH2AX, red) and (**b**) CD56+ NK cells (CD56, red; γH2AX, green) of patients following total body radiation. Representative images are shown. (**a**) B cells: 20 CD19+ B cells were quantified per sample. No CD19+ B cells could be found on the slide at d3 (n.d., not detectable). In general, there were only small amounts of γH2AX foci induced in B cells, with a maximum of 1 focus per cell at day 2 (t-test, *p < 0,05). (**b**) NK cells: At d1, d2 and d3 only very low numbers of CD56+ NK cells could be found on the slides because of high cytotoxicity. The surviving cells showed an increase of γH2AX foci with cumulative dosage.

To assess the DSB level in blood cells following radiation in vivo, we immobilised PBMCs 12–14 h after fractionated TBR (2 × 2 Gy) on a glass slide and stained them with a surface marker for T cells (CD3+) and monocytes (CD14+) and simultaneously with γH2AX. Representative images are shown in Fig. 5a. The quantification revealed that following cumulative doses of up to 12 Gy the γH2AX foci level gradually increased in T cells more extensively than in monocytes (Fig. 5b). In granulocytes ex vivo we could not observe any γH2AX staining, even after 12 Gy TBR (Fig. 5a), which is in line with the data obtained in vitro (Fig. 3a). We also assessed CD19+ B cells and CD56+ NK cells as to γH2AX foci formation upon irradiation of patients (Fig. 6). In B cells, γH2AX foci were hardly detectable 1 day after TBR and showed a significant increase 2 days later (cumulative 8 Gy), but on day 3 of irradiation (cumulative 12 Gy) cells with foci were not detectable anymore, indicating efficient elimination of damaged B cells (Fig. 6a). In contrast to this, in NK (CD56+) cells the surviving population displayed a stepwise increase in the γH2AX foci level in the irradiated patient (Fig. 6b), indicating that in the surviving NK cell pupulation DNA damage accumulates following repeated radiation treatment. Whether these cells undergo apoptotic death at later times or have the ability to survive in the presence of DNA damage remains to be determined.

## Discussion

Despite the frequent use of IR in therapy and diagnostics and the unavoidable exposure to external radiation, our knowledge on radiation sensitivity of the subsets of human blood cell populations is limited and published data are heterogeneous^6,15,16^. This is because different experimental settings were used over the years in different laboratories. Thus, radiation sensitivity of lymphocytes was determined by the neutral and alkaline comet assay, the viability assay or the clonogenicity assay, each of them gives a different outcome. In most cases, phytohemagglutinin (PHA)-stimulated lymphocytes were used. Interestingly, there is a significant difference in the sensitivity of proliferating versus non-proliferating lymphocytes to IR and chemical genotoxicants, with non-proliferating (non-stimulated) lymphocytes being more radiation-sensitive than proliferating (CD3/CD28-stimulated) lymphocytes obtained from the same donor^8^. For a comparison of different blood cell populations non-proliferating cells should be investigated because a) myeloid cells (monocytes, macrophages, DCs) in vitro do not proliferate, b) most of the lymphoid cells in the peripheral blood in vivo do not proliferate (unless they were activated and expand during infection).

To compare the radiosensitivity of the different hematopoietic cell populations, we set out to determine the responses of different cell types obtained from peripheral blood of healthy volunteers in the same setting and under the same experimental conditions (without PHA or CD3/CD28 stimulation) of lymphoid and myeloid cells (freshly isolated from buffy coats) ex vivo. Our results show that T and B cells are highly sensitive, starting to undergo apoptosis following irradiation with a dose as low as 0.125 Gy. Importantly, in the low dose range, the apoptosis yield increased linearly up to a saturation level of about 50% with a dose of 1 Gy. We did not observe strong differences between the subsets of T cells. In the apoptosis assay (AV+), Th cells seemed to be the most sensitive fraction, showing after irradiation with 0.5 Gy an apoptosis level of about 30%, compared to Treg, NK and B cells with 20–25% (Fig. 1a,c). However, because of the high interindividual variability, the differences between the T cell subsets were statistically not significant (see Supplement Fig. S1b). In both the AV+ and subG1 assay, CTL were highly sensitive, achieving apoptosis rates similar to Th cells (Fig. 1a). Significantly elevated apoptosis rates were observed already with a dose of 0.125 Gy in the different subsets of T cells, B and NK cells, as well as CD34+ progenitor cells. Interestingly, in the dose range investigated, we did not observe a no-effect threshold indicating the apoptosis programme is ready to be activated (stochastically) in a fraction of cells already at a low DNA damage level.

The high sensitivity of peripheral blood lymphocytes to radiation is in accordance with data obtained by Nakamura et al., who showed already in 1990 that CD4^+^ and CD8^+^ T cells and unsorted lymphocytes die dose dependently, with about 50% death fraction following 2 Gy^17^, and Pugh et al. concluded that lymphocytes are highly sensitive to the lethal effects of IR with apoptosis playing a major role^18^. It is important to note that the differences between T and B cell populations were not clearly significant, which confirms a previous study by Horn et al., supporting their conclusion that “for biodosimetry this makes analyzing individual subsets unnecessary”^16^. We should note that the frequencies of apoptosis of PBLs and their subsets measured in our assays are higher than reported by Bordon et al.^19^ and Horn et al.^16^. They observed highest frequencies 3 days after radiation treatment (about 40% after 0.5 Gy^16^). Furthermore, Horn et al.^16^ defined apoptotic cells by flow cytometry scatter signals in combination with caspase activity whereas we used the common annexinV (AV) staining method. We did not perform time course experiments, and we have to admit that we used EDTA (instead of heparin) for blood sampeling, which might have an effect on the viability and background apoptosis frequencies^20^. Very low apoptosis frequencies were reported by Falcke et al., which might be explained by different gating strategies and subgrouping into early and late apoptosis/necrosis^21^. It is important to note that TBR with single doses of 2 Gy is commonly used to eradicate nearly completely T and B cells prior to stem cell transplantation, which supports the view that PBLCs are more radiation sensitive than many other targets in the body.

Compared to cells of the lymphoid lineage, monocytes isolated from peripheral blood are clearly more radiation-resistant. Thus, with a dose of 2 Gy about 10% of monocytes were AV+ compared to 30–50% of lymphoid cells and CD34+ progenitor cells (Fig. 1a). The high radiation resistance of monocytes compared to lymphocytes is in accordance with a report of another group^21^. Even more resistant than monocytes are macrophages and iDCs, and thus the myeloid lineage appears to be highly radiation resistant compared to the lymphoid lineage. Previously, we reported that monocytes are limited in DNA repair and more radiation-sensitive than macrophages and DCs, which were derived from them by cytokine maturation^7^. This finding is confirmed by the data reported here. Thus, with doses up to 2 Gy macrophages and iDC hardly underwent apoptosis (Fig. 1a). Monocytes do not express the repair proteins XRCC1, ligase III and PARP-1, which are required for non-homologous end-joining (B-NHEJ) of broken DNA ends^22,23^. Due to the limited repair capacity, they display a lower DNA repair rate, which becomes obvious especially at high dose levels when the residual repair activity is exhausted^7,8^. Monocytes are also impaired in base excision repair (BER), which impacts their sensitivity to alkylating agents^12,13^. Whether the BER deficiency due to downregulation of XRCC1 (which is required for the ligation step following removal of the damaged base) contributes to the radiation sensitivity of monocytes (compared to macrophages and DCs) is an open question. In this context, it should be stressed that all cell types in this study were not stimulated (except CD34+) and therefore are not proliferating. Thus, possible interference of DNA repair intermediates with the replication machinery, as proposed previously^24^, does not play a role in non-stimulated hematopoietic cells. Interestingly, if lymphocytes were stimulated to proliferate, they became less radiation-sensitive, which goes along with downregulation of ATM expression and signalling^8^.

If monocytes are impaired in DNA repair and, on the other hand, non-stimulated lymphocytes are repair competent, why are (non-stimulated) lymphocytes more radiation-sensitive than monocytes? It is reasonable to hypothesize that lymphocytes are primed to undergo apoptotic death, which is their normal way of elimination that occurs permanently in blood and tissues^25^. Obviously, even a small amount of DNA damage seems to be sufficient to activate the DNA damage response and the downstream apoptosis pathway^26^. In contrast, in monocytes, Mph and iDC the death receptor pathway is not spontaneously activated^7^ and, despite a high level of accumulated DNA damage, cells are not primed to undergo apoptosis.

To address the question whether the high radioresistance of monocytes compared to lymphocytes can also be observed in vivo, we examined blood samples from patients who received total-body radiation (TBR) (2 × 2 Gy per day, cumulative 12 Gy). We observed a strong decline in the amount of lymphocytes, but not of monocytes (relative to PBMC). Even after cumulative 12 Gy, monocytes were not completely depleted, which supports the notion that monocytes are significantly more radioresistant than B, T and NK cells. Thus, the in vivo data basically confirm what we observed in vitro.

Previously, we showed that monocytes are vulnerable to ROS, going to death by poisoning due to their own ROS burst or if macrophages in their vicinity were activated for ROS production^27^. This scenario, termed "*killing in trans*", was proposed to take place in the inflamed tissue, regulating the amount of inflammation-driving immune cells such as monocytes, macrophages and DC^27^. The high radioresistance of monocytes compared to lymphoid cells is not in contradiction to this concept, which rests on local cell–cell interactions. In cancer radiotherapy, tumour-associated monocytes, macrophages and DCs, which are important for activating a tumour-specific immune response, are likely to survive radiation exposure with 2 Gy. Although we do not know the effect of fractionated doses, it is conceivable that the cells survive fractionated doses as well.

Monocytes are generated in the bone marrow from hematopoietic stem cells, which are radiation-sensitive^6^. They maturate in the bone marrow and thereafter are released in the peripheral blood where they stay for a period of 2–5 days until they migrate into the tissues^28^. In contrast, lymphocytes are long-living cells, remaining in the body up to 2 years, naive T cells even up to 9 years^29^. Depletion of hematopoietic stem cells during TBR is therefore expected to cause a decline in the number of monocytes at late times after radiotherapy. Indeed, we observed some fluctuation, also reported by others^30^, which may result from compensative pool-filling^6^, but we did not observe strong monocyte depletion compared to T cells, which were strongly depleted in the period up to 3 d after the onset of TBR. Nevertheless, we cannot exclude that depletion of monocytes occurs at a later stage, causing a decline of the innate immune response at late times after TBR. The data are in line with radiation effects observed in rats upon low dose irradiation. Already 0.3 Gy resulted in reduction in blood cell counts one day after irradiation^31^.

We also investigated the response of granulocytes (the major fraction is neutrophils). Similar to our previous report^9^, granulocytes did not display γH2AX foci formation, supporting the notion that in the final stage they are impaired in DNA damage signalling. They start to die during the cultivation period. Thus, the high spontaneous apoptosis level made it impossible to determine a radiation-induced response. Of note, in total blood counts after TBR, neutrophiles did not decline during the radiation period, indicating they are highly radiation resistant or are permanently replaced by a radioresistant precursor.

High dose radiotherapy (1.8–2 Gy) used for cancer treatment is thought to kill tumour cells directly and, indirectly, through activating a trans-acting immune response^32^. Moreover, low dose radiation with a single dose of 0.5 Gy has been demonstrated to be effective in the treatment of rheumatoid arthritis associated with pain and inflammation^33^. Although macrophages (M1) are drivers in this process through proinflammatory cytokine secretion, they can hardly be considered as a target of inflammatory radiotherapy because of their high level of radioresistance. We should note, however, that we cannot exclude the possibility that they are functionally impaired following radiation treatment, which needs to be investigated. With the published data at hand, it is reasonable to consider CTL and other lymphoid cell types as targets of low dose radiotherapy^6,34,35^. This notion is supported by this study revealing a high radiosensitivity of T cells, which will inevitably cause their depletion in the inflamed tissue. We should also note that DC vaccination in combination with radiotherapy might be an effective strategy since tumor-infiltrating radioresistant DC are likely to survive treatment and able to activate the antitumoral immune response within the irradiated tumor tissue.

## Material and methods

### Cell isolation and culture

*Buffy Coat*: Peripheral blood mononuclear cells (PBMC) were separated by Histopaque (Histopaque 1077, Sigma-Aldrich) density gradient centrifugation from leukocyte rich plasma of healthy human donors provided by the blood bank of the University Medical Center Mainz. PBMC were cultivated in six-well Corning plates with RPMI and 1.5% autologous serum. Autologous serum was separated during density gradient centrifugation and collected afterwards. After 30 min of cultivation (37 °C at 5% CO_2_), monocytes attached to the bottom of the plates, whereas peripheral blood lymphocytes (PBL, consisting mainly of T cells) remained in the supernatant^8^. Monocytes were cultivated in X-VIVO-15 medium (Bio Whittaker) supplemented with 1.5% autologous serum and differentiated into macrophages (Mph) or immature dendritic cells (iDC) with GM-CSF (Bayer Healthcare) and respectively IL-4 as described previously^7,12^. Natural Killer (NK) cells, B cells and stem/progenitor cells were isolated from PBMC with magnetic bead coupled antibodies (Miltenyi Biotec, Bergisch-Gladbach, Germany) targeting CD56, CD19, CD14 and CD34. The setting of gates for analysing annexin V within the different blood cells is shown in Supplement, Figs. S3 and S4. The isolation of Treg cells, Th cells and CTLs with magnetic beads was described previously^8^. CD34 progenitor cells were cultivated and expanded in Stem MACS HSC Expansion Medium XF with Stem MACS HSC Expansion Cocktail (both Miltenyi Biotec) for 6 days before irradiation and cell death analysis. For DNA repair kinetics, the cells were irradiated directly after purification. Granulocytes (the major fraction is neutrophils) were isolated by the OptiPrep (Axis-shield) density centrifugation method based following the manufacturer’s protocol for leukocyte rich plasma (application sheet C11). During centrifugation, the white blood cells were separated into two rings. The upper ring contains mononuclear cells (PBMC) and the lower ring polymorphonuclear cells (granulocytes).

*Whole blood*: PBMC and granulocytes were purified from whole blood by density centrifugation using Polymorphprep (Axis-shield). 5 ml Polymorphprep was overlayed with 5 ml whole blood (sample from patients) and centrifuged 30 min without break. As to the OptiPrep method, PBMC (upper ring) and granulocytes (lower ring) were separated into two distinct bands.

### Irradiation

Purified blood cells from buffy coat were irradiated within a gamma cell irradiator 2000 (Cs-137 source, Molsgaard Medical, Denmark) in a dose equivalent time frame. The dose rate was 2.52 Gy/min.

### Cell death analysis

Cell death was determined by the annexin V-assay and subG1 staining by flow cytometry (FACSCanto II, BD Biosciences) as previously described^8^. For the annexin V-assay, the cell pellet was resuspended in 25 µl 1 × annexin V-binding buffer (pH 7.4, 10 mM HEPES, 140 mM NaCl, 2.5 mM CaCl_2_, 0.1% bovine serum albumin) including 1.25 μl annexin V-FITC (Miltenyi Biotec) and incubated for 20 min in the dark at room temperature. Th and CTL were stained additionally with CD3-PE and CD4-VioBlue and CD8-APC respectively within the PBMC population as shown in Fig. S1 with appropriate compensation steps when they were not pre-purified by magnetic beads. For determination of DNA fragmentation by the subG1-assay, the cells were fixed and permeabilised in ice-cold 80% ethanol by storing for 1 h up to 2 weeks at − 20 °C. The suspension was centrifuged and the supernatant removed. The pellet was resuspended in 333 μl PBS+ 1 μl RNase (stock 10 mg/ml). After 1 h at room temperature, 164 μl PI (stock 50 μg/ml) was added to the solution and samples were stored on ice until analysis was performed by flow cytometry. The FACSDiva (BD) software and the flowing software 2 were used for data analysis. The radiation-induced effect on cell death was calculated by subtracting the basal level of the untreated control from the irradiated sample. For the depletion analysis of CD3+ and CD14+ cells by flow cytometry, the antibodies CD3-PE and CD14-VioBlue (both Miltenyi Biotec) were used.

### Immunohistochemistry

The method of γH2AX foci staining and determination of DNA repair kinetics in peripheral blood lymphocytes used in our laboratory was in detail previously described^36^. Two different imaging systems were used. The LSM 710 system (Carl Zeiss, Oberkochen, GER) was used along with the ZEN software for Laser Scanning Microscopy. The Metafer slide scanning system is applied to the Axio Imager M1 (Carl Zeiss, Oberkochen, GER) operated with the Metafer4 software (MetaSystems, Altlussheim, GER). Cells for LSM analysis (double staining with CD markers) were permeabilized by treatment with methanol:aceton (7:3) for 6 min at -20 °C. After washing 2 times with PBS (carried out after each fixation/permeabilisation step), the samples were fixed with 2.5% paraformaldehyde for 10 min at room temperature. Samples for Metafer slide scanning were fixed with 4% paraformaldehyde for 10 min at room temperature and after PBS washing steps incubated with ice-cold methanol for 10 min at − 20 °C. The samples were blocked with 10% goat normal serum in PBS for 30 min (for Metafer slides additional 0.1% Triton X-100 was included). First antibodies were incubated for 1 h diluted 1:100 up to 1:500 in 1% BSA in PBS. Second antibodies were diluted 1:300 (Alexa Fluor488) and 1:500 (Cy3) in 1% BSA in PBS and samples incubated for 1 h. Slides for LSM analysis were additionally stained for 15 min with ToPro3 (Invitrogen, Oregon, USA) for nucleus staining. LSM slides were mounted in Vectashield medium (Burlingame, USA), Metafer slides in Vectashield medium with DAPI and sealed with nail polish. Cells analysed by the Metafer slide scanning system were purified with magnetic beads or, in the case of macrophages, differentiated from monocytes prior irradiation and γH2AX staining. The LSM 710 was used when cells were additionally stained with CD antibodies for blood cell phenotyping. The following primary antibodies were used: anti-CD3 (MCA1477), anti-CD56 (CA2693GA), anti-CD19 (MCA2454) (all from AbD Serotec, Bio-Rad München), anti-CD14 (63,319), anti-CD34 (8536), anti-CD64 (119,843) (all from Abcam, Cambridge UK) and anti-CD15 (555,400, BD Pharmingen). The secondary antibodies used were F(ab’2) goat anti-mouse IgG (H + L) coupled with Alexa Fluor 488 (A11017), F(ab’2) goat anti-rabbit IgG (H + L) coupled with Alexa Fluor 488 (A11070) (both Life Technologies, Carlsbad, CA), goat anti-mouse IgG (H + L) coupled with Cy3 (115,165,146 Dianova, Hamburg, GER) and goat anti-rat IgG (H + L) coupled with Cy3 (112,165,143, Jackson Immuno, Cambridgeshire, UK). Metafer slides were counted with ImageJ using an appropriate macro. Foci recorded by the LSM 710 were counted by eye or analysed by measuring the mean intensity of the nuclear γH2AX staining using ImageJ software.

### Western blot

Protein detection was performed as described in Ponath et al*.*^27^. Cell pellets were lysed with RIPA buffer (50 mM Tris, 150 mM NaCl, 1 mM EDTA, 1% NP-40, 0.5% sodium deoxycholate, 0.1% SDS, 0.1% sodium azide, 1 mM PMSF, 2 mM sodium orthovanadate, 2 mM DTT and freshly added 1 × protease inhibitor, pH 8.0). Samples were snap-frozen and thawed on ice twice before they were sonicated with 2 × 5 pulses. Samples were centrifuged at 13,000 *g* for 10 min at 4 °C. The protein concentration of the supernatant was measured via the Bradford method; 50–100 µg of protein were used for loading. Samples were boiled in loading buffer for 5 min at 56 °C for large proteins (> 140 kDa) and at 95 °C for small proteins. SDS-PAGE was performed at 60 V for the stacking gel and increased to 100 V when samples entered the running gel. Proteins were transferred to nitrocellulose membranes at 300 mA for 90 min or at 100 mA overnight at 4 °C using a buffer composed of 100 ml 5 × Laemmli buffer (30 g Tris, 144 g glycine in 1 l ddH_2_O), 200 ml methanol, 10 ml 10% SDS, and 1 l ddH_2_O. Western blot membranes were blocked in 5% BSA-PBS or 5% dry milk in TBS with 0.1% Tween20. The following primary antibodies were used: anti-ligase III (BD Transduction Laboratories), anti-XRCC1 (Abcam), anti-PARP1 (c-II10, a kind gift from Prof. A. Bürkle from the University of Konstanz, GER), anti-GAPDH (Santa Cruz Biotechnology, Heidelberg, GER). Detection was performed by the Odyssey imaging system (LI-COR Biosciences, Bad Homburg, GER) with secondary antibodies coupled to infrared dyes (IRDye 800CW and IRDye 680).

### Patients

Experiments with primary patient blood samples were approved by the ethic committee of the Landesärztekammer Rheinland-Pfalz (No. 837.270.05; 4928). They were conducted in accordance with the declaration of Helsinki. All patients received allogeneic stem cell transplantation as consolidation therapy for acute leukaemia and were in complete remission at the time point of the study. After informed consent was obtained, 10 ml EDTA peripheral blood was taken at each indicated time point and immediately transported to the laboratory for further experimental analyses.

### Statistics

Statisical analysis (Two-Way ANOVA, Tukey; One-Way ANOVA, Dunnett; and t-test) was performed with GraphPad Prism (GraphPad Software, San Diego, CA).

## Supplementary Information

Supplementary Figures.
