# Supplementary material for: Comparison of DNA repair and radiosensitivity of different blood cell populations
Source: Sci Rep. 2021 Jan 28;11:2478. doi: 10.1038/s41598-021-81058-1 (PMC7843614; doi:10.1038/s41598-021-81058-1)
Supplement: Supplementary file 1 — Supplementary Figures. [file 41598_2021_81058_MOESM1_ESM.pdf]

Supplementary material

Comparison of DNA repair and radiosensitivity of different blood cell populations

Daniel Heylmann[1#], Viviane Ponath[1$], Thomas Kindler[2] and Bernd Kaina[1*]

[1]Institute of Toxicology, University Medical Center, Mainz and
[2]Department of Medical Oncology and Pneumology, University Medical Center, Mainz, Germany

a

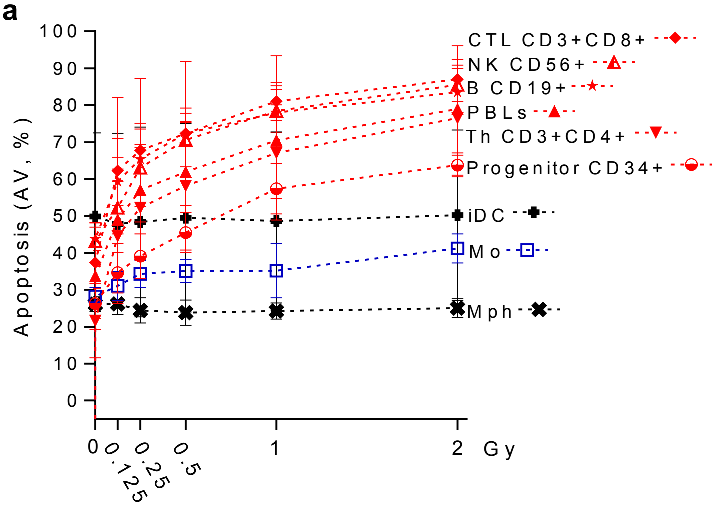

b

0.125	0.25	0.5	1	2 [Gy]
*CTL ↔ DC	***CTL, Th ↔ DC, Mph	***Th, CTL ↔ Mph, DC	***Th, CTL, PBL, B, NK, CD34 ↔ Mph, DC	***Th, CTL, PBL, B, NK, CD34 ↔ Mph, DC
		**Th ↔ Mo	***Th, CTL ↔ Mo	***Th, CTL, PBL ↔ Mo
		**B, PBL ↔ Mph, DC	**PBL ↔ Mo	**NK ↔ Mo
		**NK ↔ Mph	*B, NK ↔ Mo	*B ↔ Mo
		*CTL ↔ Mo		
		*NK ↔ DC		

c

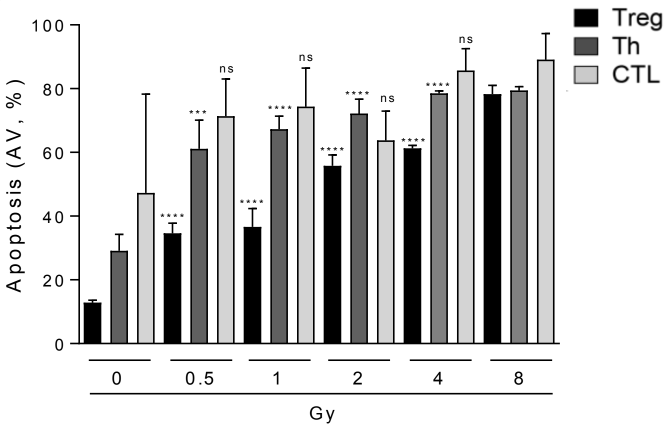

d

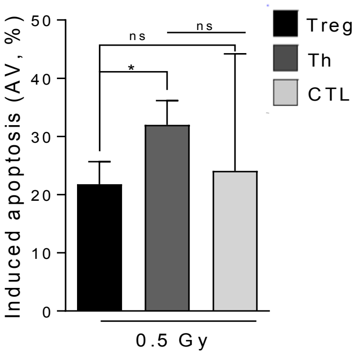

Figure S1

a) Absolute apoptosis frequencies from experiments shown in Fig.1a. **b)** Statistical analysis of Fig.1a (Two-Way ANOVA (Tukey), $*p < 0.05$, $**p < 0.01$, $***p < 0.001$, $n = 3$) **c)** Absolute apoptosis frequencies from experiments shown in Fig.1c (One-Way ANOVA, Dunnett, compared to 0 Gy, $***p < 0.001$, $****p < 0.0001$, $n = 3$, 0 – 4 Gy; $n = 2$, 8 Gy). **d)** Induced radiation response from Fig.1c only shows significant differences between Treg and Th after 0.5 Gy (t-test).

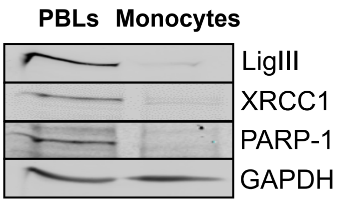

Figure S2
Western Blots for ligase III, XRCC1 and PARP-1. GAPDH served as loading control.

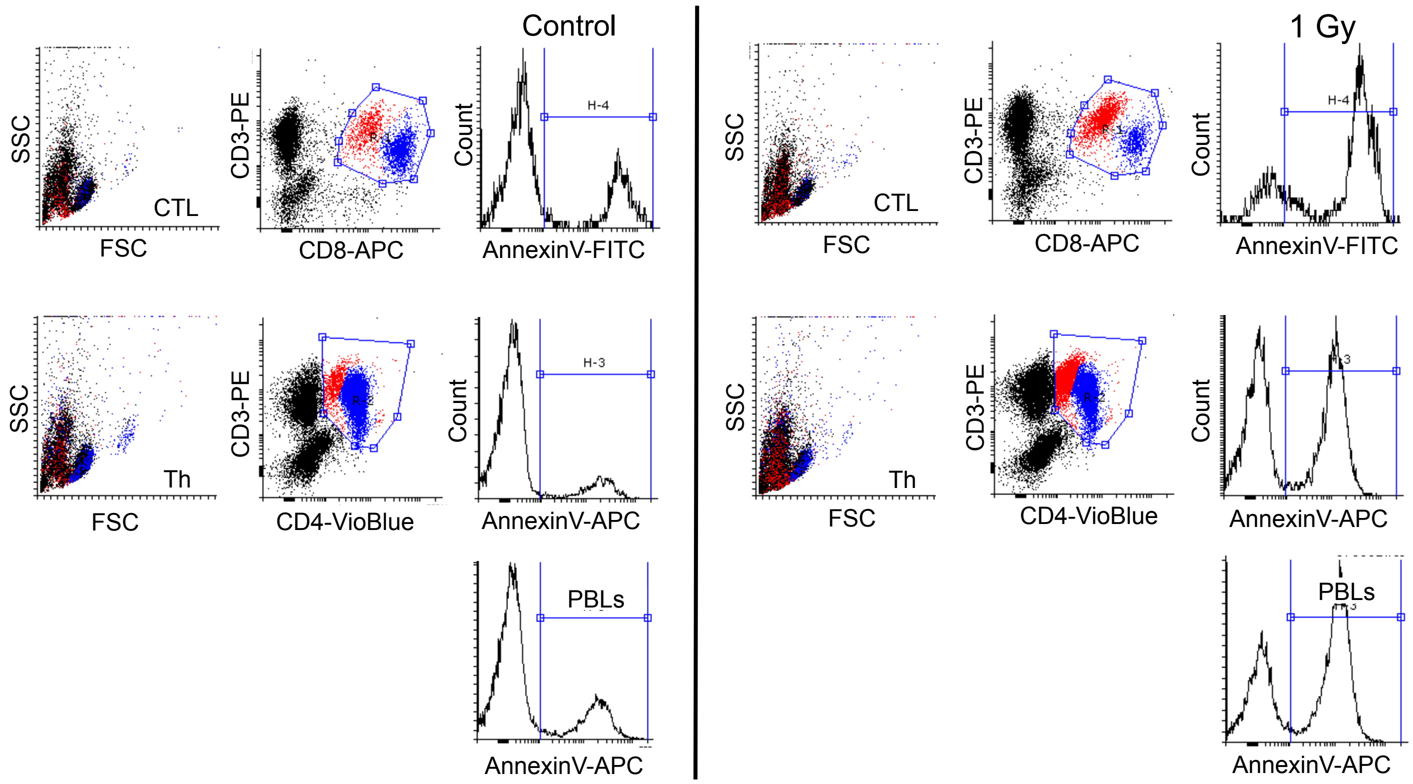

Figure S3
Dotblots and histograms of PBMCs stained for annexinV, gated for CD3 and CD4 respectively CD8. Analysis performed by flow cytometry. Left side non-irradiated (control), right side 24 h after irradiation with 1 Gy. The radiation induced shift of CD8 or CD4 positive cells (blue) to the left (red) implies a loss of the surface marker, which goes along with increased cell death. SSC (Sideward Scatter), FSC (Forward Scatter).

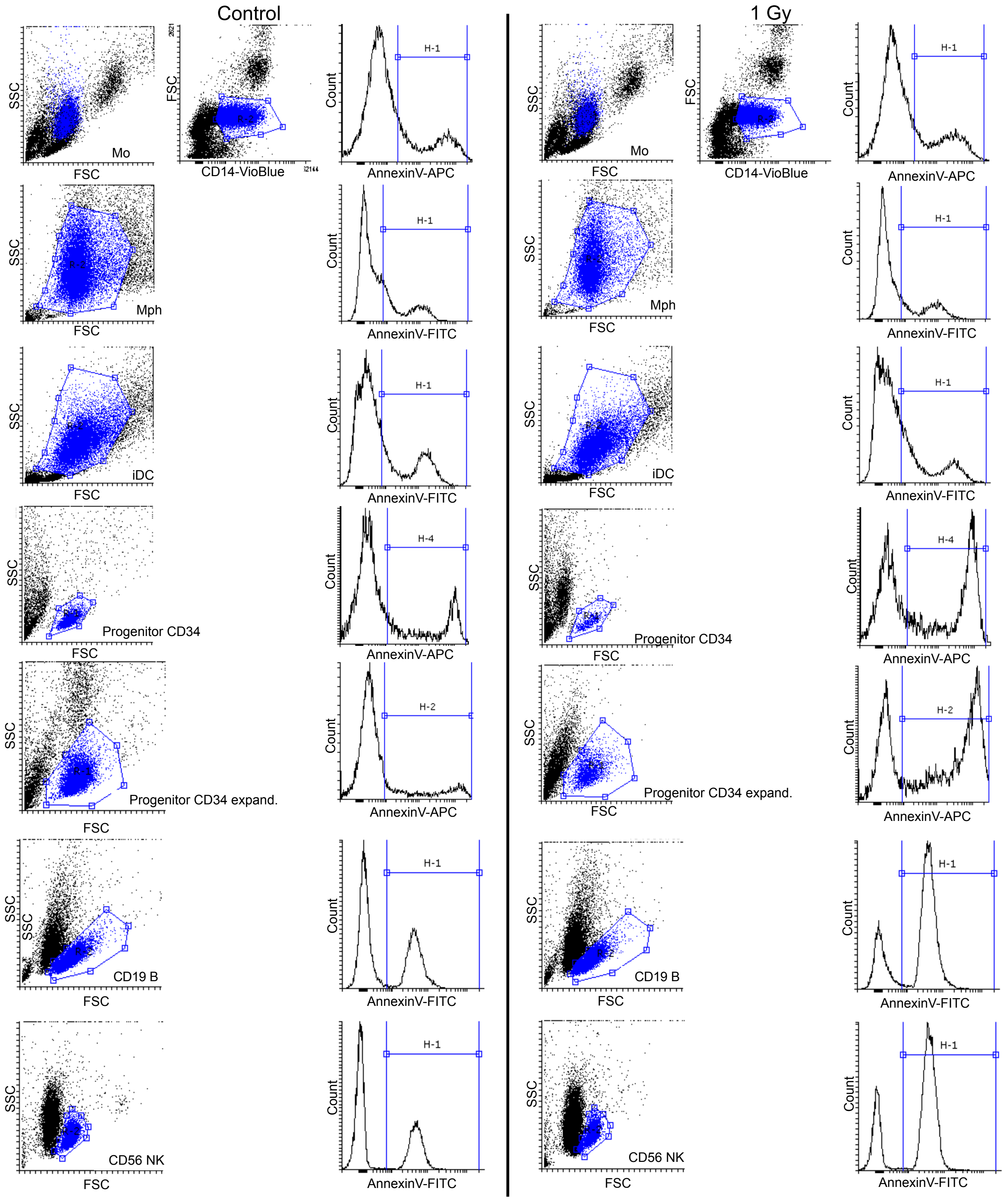

Figure S4
SSC and FSC dotblots of non-irradiated (control) and irradiated (24 h after 1 Gy) purified blood cells with corresponding histograms revealing annexinV-staining. Monocytes were additionally stained with CD14-VioBlue and gated for annexinV.
